# mRNA-Lipid Nanoparticle-Mediated Reprogramming and Standard Sendai Virus Reprogramming: Generation of iPSCs and iPSC-Derived Cardiomyocytes

**DOI:** 10.3390/ijms27083588

**Published:** 2026-04-17

**Authors:** Marlon DeBose, Jonathan Choi, Dingqian Ding, Anna G. Griggs, Elisa Marie Gollatz, Evan Scislowicz, Adriana Harbuzariu, Ilanit Itzhaki

**Affiliations:** 1 Pediatric Children’s Heart Center, Department of Pediatrics, School of Medicine, Emory University, Atlanta, GA 30322, USA; mdebose@emory.edu (M.D.); jonathan.choi@emory.edu (J.C.);; 2 Children’s Heart Research and Outcomes (HeRO) Center, Children’s Healthcare of Atlanta and Department of Pediatrics, School of Medicine, Emory University, Atlanta, GA 30322, USA; 3 School of Medicine, Emory University, Atlanta, GA 30322, USA; aggrigg@emory.edu (A.G.G.); elisa.marie.gollatz@emory.edu (E.M.G.); adriana.harbuzariu@emory.edu (A.H.)

**Keywords:** Sendai virus, mRNA, lipid nanoparticles

## Abstract

For over a decade, non-integrating Sendai virus vectors have been the gold standard for induced pluripotent stem cell (iPSC) reprogramming. However, as the field shifts toward regenerative and precision medicine and large-scale biorepositories, Sendai virus workflow necessitates dedicated viral-clearance testing, specialized manufacturing controls, and heightened regulatory oversight, leading to increased cost. While mRNA-based reprogramming offers a non-viral alternative, traditional mRNA delivery methods like electroporation are often physiologically disruptive. This study evaluates an mRNA-reprogramming platform that delivers lipid nanoparticles (mRNA-LNPs) via receptor-mediated endocytosis. By utilizing both Sendai virus and mRNA-LNP approaches to reprogram PBMCs from the same donor, we established a genetically identical starting point. Results demonstrate that mRNA-LNP-reprogrammed iPSCs maintain genomic integrity, retain the donor *KCNH2* c.2398+5G>T variant, and exhibit characteristic colony morphology, pluripotency markers, and trilineage differentiation capacity consistent with the Sendai-reprogrammed counterparts. The mRNA-LNP-reprogrammed iPSCs differentiate into iPSC-derived cardiomyocytes presenting sarcomeric structures and electrophysiological activity, recapitulating a disease-specific phenotype. Notably, the mRNA-LNP workflow reached these milestones in significantly fewer passages than the Sendai virus workflow, markedly shortening timelines and reducing costs. These findings highlight mRNA-LNP reprogramming as a potentially attractive and effective, virus-independent platform to support future regenerative and precision medicine initiatives and scalable biobanking.

## 1. Introduction

For over a decade, Sendai virus-based reprogramming has played a central role in the field of induced pluripotent stem cell (iPSC) biology [1,2,3]. As a non-integrating, cytoplasmic RNA virus, it enables highly efficient delivery of the Yamanaka factors without altering the genome [3,4], producing iPSCs with stable pluripotency and broad differentiation potential [5,6]. Its non-integrating, transient expression system made it one of the first practical and reproducible platforms for generating iPSCs compatible with clinical-translation workflows, and it has substantially accelerated applications across disease modeling, regenerative medicine research, cardiotoxicity screening, and drug discovery [5,7,8].

These strengths, however, coexist with constraints that become more consequential when moving from research settings toward real-world iPSC regenerative therapies [9]. As iPSC-derived cardiomyocytes (iPSC-CMs) advance beyond academic use and toward applications in clinical cardiac precision medicine [10], the method used to generate the parental iPSCs becomes increasingly important. Although Sendai virus is non-integrating and typically diluted by approximately passage 10 [11], it remains a replicating vector, and rare cases of residual viral genomes have been reported up to passage 20 [4,12]. As a result, its use requires dedicated viral-clearance testing, specialized manufacturing controls, and heightened regulatory oversight [13].

These constraints become even more pronounced as the demand for large, standardized iPSC biorepositories grows. Diluting out the Sendai virus vector requires multiple early passages, which consumes valuable early-passage iPSC resources, extends production timelines, and increases demands on media, consumables, labor, and incubator capacity [14]. Each additional passage increases the risk of spontaneous differentiation and contamination events and accelerates progression toward later passages where karyotypic stability becomes a more prominent concern [15]. Together, these effects reduce throughput and delay downstream banking and differentiation. When scaled across hundreds of donor lines, these cumulative burdens increase cost, slow expansion, and complicate the workflows needed to generate iPSCs suitable for large-scale distribution and eventual clinical translation [16,17].

As interest in non-viral reprogramming grew, mRNA-based methods emerged as a promising alternative [18,19]. Although mRNA reprogramming offers a non-integrating, virus-independent option, the delivery formats used in many such protocols can place substantial stress on cells [19,20]. These approaches most commonly use lipid-based transfection or electroporation to introduce synthetic mRNAs [20,21]. Lipid-based transfections perturb the plasma membrane, and electroporation physically disrupts it, both of which can place strain on sensitive primary cells [22,23,24]. mRNA-lipid nanoparticles (mRNA-LNPs) were developed to address these limitations by entering cells through receptor-mediated endocytosis, a natural uptake pathway that avoids membrane disruption [25,26], while maintaining the transient, “zero-footprint” nature of synthetic mRNA, which leaves no integrating or persistent genetic material in the cell [19,27,28].

In this study, we compare Sendai virus- and mRNA-LNP-mediated reprogramming of human peripheral blood mononuclear cells (PBMCs) to evaluate whether this non-viral, transient platform can generate iPSCs that perform comparably to those produced using the established Sendai virus method. We characterize the resulting iPSCs across morphology, pluripotency hallmarks, genomic integrity, and the passage number at which Sendai virus and mRNA-LNP methods reach reprogramming and characterization milestones. To assess downstream functional performance, we differentiate iPSCs generated by each method into iPSC-CMs and examine their retention of a patient-specific variant and their ability to recapitulate the corresponding electrophysiological phenotype in vitro. By reprogramming PBMCs from the same donor using both Sendai virus and mRNA-LNP approaches, we established a genetically identical starting point that enables a direct comparison between methods. As a representative disease model with a well-defined and readily measurable electrophysiological signature, we leveraged PBMCs from a long QT syndrome (LQTS) patient to evaluate whether mRNA-LNP-derived iPSCs, like their Sendai-derived counterparts, preserve the pathogenic *KCNH2* variant and recapitulate the characteristic action potential prolongation upon differentiation [29,30,31]. Together, these assessments provide an evaluation of mRNA-LNP reprogramming across a comprehensive set of iPSC characterization assays and downstream iPSC-CM electrophysiological readouts.

## 2. Results

**Figure 1:**
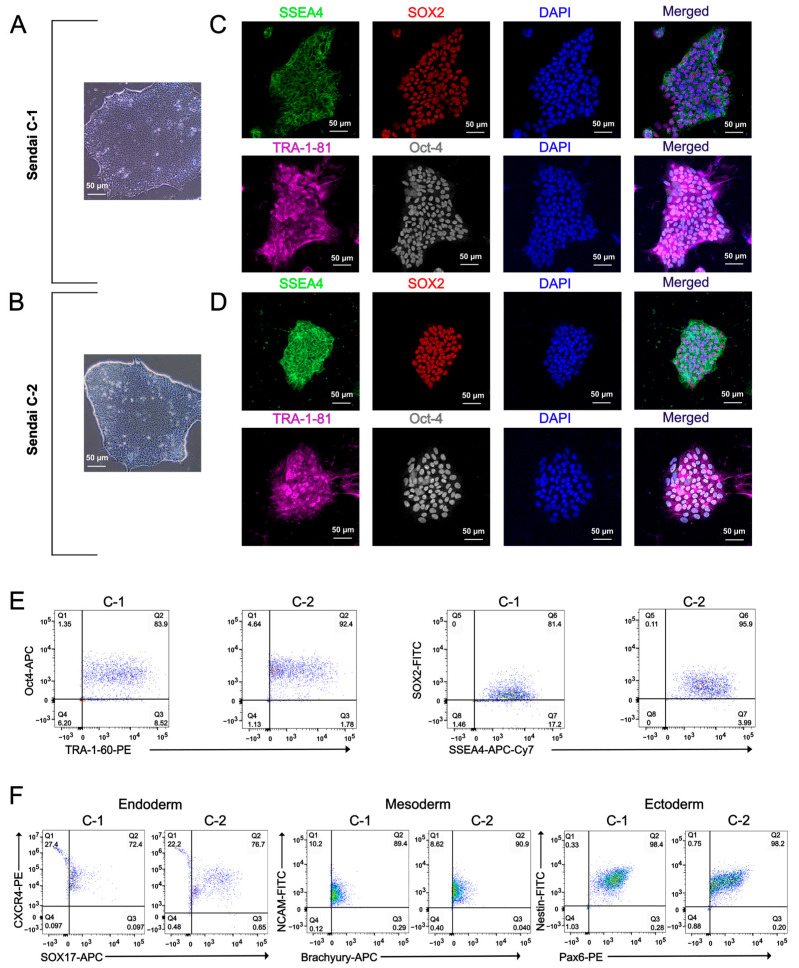
PBMCs were reprogrammed into iPSCs using a non-integrating Sendai virus method. (**A**) Light microscopy images displaying characteristic iPSC colony morphology in clone 1 (Sendai C-1) and (**B**) in clone 2 (Sendai C-2). (**C**) Immunocytochemistry displaying the expression of pluripotency markers SSEA4, SOX2, TRA-1-81 and Oct-4 in clone 1 and (**D**) clone 2. (**E**) Flow cytometry analysis demonstrating the expression of Oct-4, TRA-1-60, SOX2, and SSEA4 markers for both clones. (**F**) Demonstrated pluripotency across all three germ layers: endoderm, mesoderm, and ectoderm for both clones.

**Figure 2:**
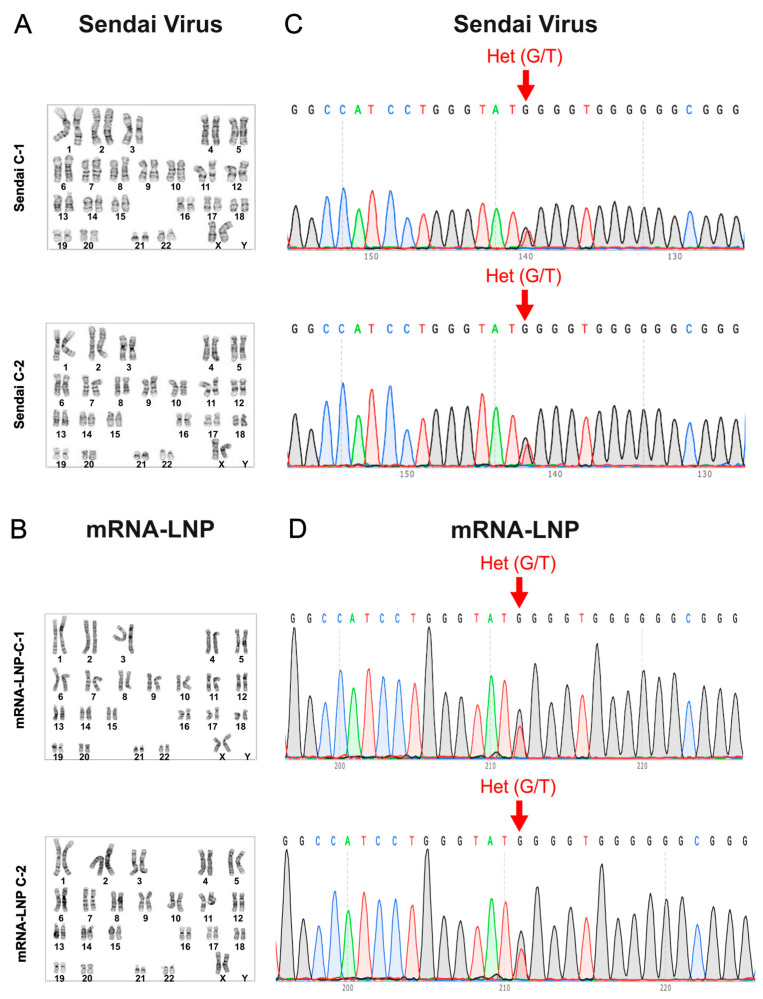
Genomic Integrity and Variant Verification. (**A**) Both Sendai virus and (**B**) mRNA-LNP reprogrammed iPSCs exhibit a normal karyotype. (**C**) Both Sendai virus and (**D**) mRNA-LNP reprogrammed iPSCs harbor the *KCNH2* variant (c.2398+5G>T), as verified by Sanger sequencing. Each assessment was repeated in two independent clones (C-1 and C-2) for each reprogramming method.

PBMC reprogramming into iPSCs using the non-integrating Sendai virus method has been one of the most widely adopted and reliable approaches for generating iPSCs over the past decade [1,2,3]. This method has supported advances in human-based and patient-specific translational disease modeling, regenerative applications, cardiotoxicity screening, and drug discovery, yielding high-quality and genetically stable iPSC lines [32,33]. Here, we present the characterization and pluripotency validation of iPSCs generated from PBMCs using the Sendai virus reprogramming method. Sendai virus-mediated reprogramming of PBMCs resulted in iPSC colonies that displayed characteristic morphology under light microscopy, forming dense iPSC colonies with well-defined borders (Figure 1A,B). Immunocytochemistry demonstrated the expression of the pluripotency markers SSEA4, SOX2, TRA-1-81, and Oct-4 (Figure 1C,D). Flow cytometry further showed expression of Oct-4, TRA-1-60, SOX2, and SSEA4 (Figure 1E). To functionally validate pluripotency, we assessed trilineage differentiation capacity. The iPSCs successfully generated derivatives of endoderm, mesoderm, and ectoderm, confirming their ability to differentiate into all three germ layers (Figure 1F). To evaluate whether reprogramming affected genomic integrity, the resulting iPSCs were examined for chromosomal abnormalities. The iPSCs exhibited a normal karyotype (Figure 2A), confirming that the reprogramming process did not introduce detectable chromosomal alterations. In addition, the LQTS type 2 (LQT2)-associated *KCNH2* c.2398+5G>T variant carried by the patient was preserved in the iPSC lines, as confirmed by Sanger sequencing (Figure 2C). These experiments were conducted on two independent clones.

**Figure 3:**
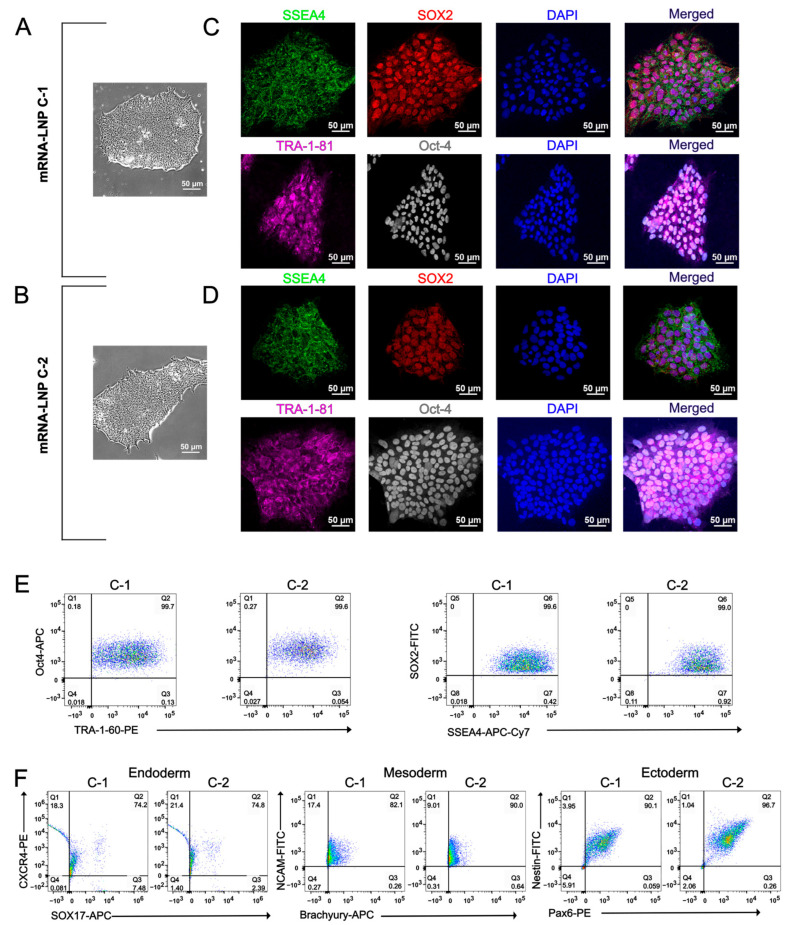
PBMCs were reprogrammed into iPSCs using the mRNA-LNP method. (**A**) Light microscopy images displaying characteristic iPSC colony morphology in clone 1 (mRNA-LNP C-1) and (**B**) in clone 2 (mRNA-LNP C-2). (**C**) Immunocytochemistry displaying the expression of pluripotency markers SSEA4, SOX2, TRA-1-81 and Oct-4 in clone 1 and (**D**) clone 2. (**E**) Flow cytometry analysis demonstrating the expression of Oct-4, TRA-1-60, SOX2, and SSEA4 markers for both clones. (**F**) Demonstrated pluripotency across all three germ layers: endoderm, mesoderm, and ectoderm for both clones.

In parallel, iPSCs generated from PBMCs using the mRNA lipid nanoparticles (mRNA-LNP) reprogramming method underwent the same characterization and pluripotency validation workflow. mRNA-LNP-mediated reprogramming produced iPSC colonies with morphology indistinguishable from those generated by the Sendai virus method, forming compact colonies with distinct borders under light microscopy (Figure 3A,B). Immunocytochemistry confirmed expression of the pluripotency markers SSEA4, SOX2, TRA-1-81 and Oct-4 (Figure 3C,D), and flow cytometry demonstrated expression of Oct-4, TRA-1-60, SOX2, and SSEA4 (Figure 3E). Functional pluripotency was further validated by successful trilineage differentiation, with the mRNA-LNP-derived iPSCs generating derivatives of endoderm, mesoderm, and ectoderm (Figure 3F). Genomic integrity was also preserved as karyotype analysis revealed no detectable chromosomal abnormalities (Figure 2B), and Sanger sequencing confirmed retention of the LQT2-associated *KCNH2* c.2398+5G>T variant in the mRNA-LNP-reprogrammed iPSCs (Figure 2D).

Importantly, while iPSCs generated using the mRNA-LNP method demonstrated pluripotency hallmarks and preserved genomic integrity, consistent with both the iPSC literature [29,30,31] and the Sendai virus-reprogrammed iPSCs in this study, the mRNA-LNP-based workflow required substantially fewer passages to reach the equivalent milestones listed in Table 1. For example, colony picking was completed by passage 10 for Sendai virus-reprogrammed iPSCs and by passage 2 for mRNA-LNP-reprogrammed iPSCs. iPSC characterization (iPSC colony morphology, markers of undifferentiated state, and trilineage differentiation assessments) was performed as early as passage 11 for Sendai virus-reprogrammed iPSCs and passage 3 for mRNA-LNP-reprogrammed iPSCs, with genomic characterization (karyotyping and STR analysis) conducted as early as passage 11 versus passage 4, respectively. Because the mRNA-LNP passaging workflow reached the characterization milestones at a lower passage number, and each passage used the same media type and volume and the same number of consumable units (plates, serological pipettes, etc.), this resulted in a 63.6% reduction in the media volume and consumables required for passaging per clone compared with the Sendai passaging workflow. Given an approximate five-day interval between passages, these differences translated into a markedly shorter derivation timeline and tissue culture-associated costs per iPSC clone, highlighting a practical advantage of the mRNA-LNP iPSC reprogramming approach without compromising iPSC pluripotency or genomic integrity.

Sendai virus-reprogrammed iPSCs have been widely used over the past decade to generate functional iPSC-cardiomyocytes (iPSC-CMs) that recapitulate patient-specific electrophysiological phenotypes in vitro [29,30,31,32,34,35,36]. Because a central requirement of any reprogramming platform is its ability to preserve the donor’s underlying functional electrophysiological phenotype, regardless of whether the donor is healthy or carries a disease-associated variant, we designed this study to evaluate whether the mRNA-LNP reprogramming method could faithfully maintain donor-specific electrophysiological phenotype through reprogramming and cardiac differentiation. Long QT syndrome (LQTS) provides an especially powerful test case for this purpose, because its electrophysiological phenotype is both pronounced and quantifiable, enabling a clear assessment of whether the disease signature is faithfully recapitulated in derived cardiomyocytes. In LQTS, this signature includes the characteristic prolongation of action potential duration (APD), mirroring the QT-interval prolongation observed clinically [29,31,32,36,37,38]. We therefore intentionally selected a donor with LQTS, using the APD prolongation as a well-defined benchmark to determine whether mRNA-LNP-reprogrammed iPSCs can generate iPSC-CMs that recapitulate the donor’s functional phenotype to the same extent as those derived from the established Sendai virus method. To ensure that any observed differences reflected the reprogramming method rather than inter-individual genetic variability, we used both the Sendai virus and mRNA-LNP methods to reprogram PBMCs from the same LQTS donor, allowing a genetically identical starting point for comparison. This genetically matched consideration isolates the reprogramming approach as the primary variable while holding the donor genome constant.

**Figure 4:**
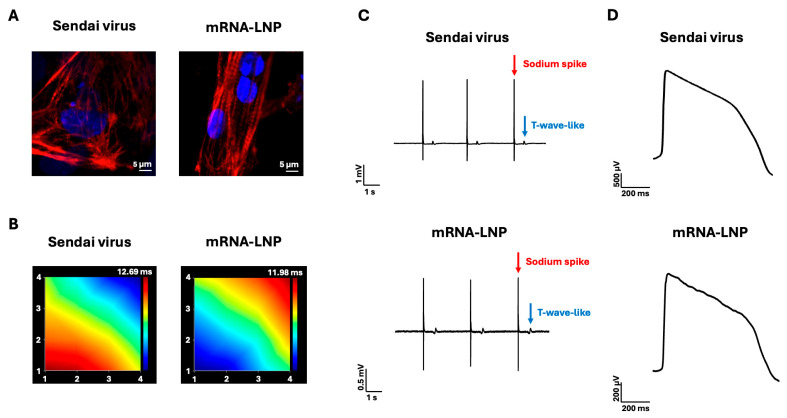
Functional Characterization of iPSC-derived Cardiomyocytes. Both Sendai virus and mRNA-LNP-reprogrammed iPSCs differentiated into iPSC-CMs exhibiting sarcomeric structures and electrophysiological activity. (**A**) Immunocytochemistry for cardiac troponin T. (**B**) Microelectrode Array signal propagation maps demonstrating electrical syncytium. (**C**) Field potential recordings depicting sodium spikes and T-wave-like waveforms. (**D**) Local Extracellular Action Potential signals.

From each method, we selected the clone with the most robust mesodermal induction, reflecting the mesodermal origin of cardiomyocytes and supporting efficient cardiac differentiation. The Sendai virus-reprogrammed clone was differentiated at passage 15 and the mRNA-LNP-reprogrammed clone was differentiated at passage 5 using an identical differentiation protocol, producing uniform, spontaneously beating iPSC-CM sheets across the wells. The resulting iPSC-CMs displayed sarcomeric structures, as demonstrated by cardiac troponin T (cTnT) immunostaining (Figure 4A). Following enzymatic dissociation, the cells were replated onto multielectrode array (MEA) plates, where they formed confluent monolayers over the electrode arrays. MEA propagation maps generated by the Axion Biosystems Maestro Edge^TM^ platform demonstrated the presence of an electrically coupled syncytium, characterized by a defined initiation site (dark blue) and a uniform unidirectional signal propagation (Figure 4B). Field potential recordings displayed characteristic depolarization (sodium spike) and repolarization (T-wave-like) components, confirming electrophysiological activity (Figure 4C). Local Extracellular Action Potential (LEAP) signals, in Figure 4D, further demonstrated a characteristic iPSC-CM ventricular-like plateau phase [39,40]. To quantify a key electrophysiological hallmark of LQTS, we measured action potential duration. Published studies have reported APD at 90% repolarization (APD90) values for “healthy” iPSC-CMs typically ranging from approximately 243 to 373 ms [37,41,42,43,44,45], whereas LQT2 iPSC-CMs consistently exhibit prolonged APD90 values, with reported ranges around 516 to 881 ms [29,30,31,36]. In our study, iPSC-CMs generated by both reprogramming approaches displayed prolonged APD90 consistent with the published LQT2 phenotype: Sendai virus-derived iPSC-CMs presented an average APD90 of 595.5 ms (n = 28; derived from 5 independent differentiations), and mRNA-derived iPSC-CMs exhibited an average APD90 of 634.3 ms (n = 28; derived from 5 independent differentiations). Together, these electrophysiological assessments indicate that the iPSC-CMs differentiated from the mRNA-LNP-reprogrammed iPSCs form a functional syncytium, generate characteristic field potentials and action potentials, and retain the capacity to recapitulate the LQTS-associated APD-prolongation phenotype, consistent with the iPSC-CM literature and aligned with observations in the corresponding Sendai virus-reprogrammed iPSC-CMs.

## 3. Discussion

Although the mRNA-LNP and Sendai virus methods rely on distinct delivery strategies, no apparent differences in the overall characteristics of the reprogrammed iPSCs or their differentiated iPSC-CM derivatives were observed. Both methods generated colonies with characteristic iPSC morphology, expressed key pluripotency-associated markers, exhibited trilineage differentiation capacity, and maintained genomic integrity. However, the mRNA-LNP method accelerated the overall workflow, reaching key reprogramming, characterization, and differentiation-initiation milestones in substantially fewer passages, and therefore in markedly less time, than the Sendai virus method. At the same time, the mRNA-LNP approach involved additional dosing steps, as the protocol requires repeated LNP administration to maintain pluripotency-factor expression, in contrast to the single-infection Sendai system.

Both reprogramming approaches ultimately supported successful iPSC differentiation to iPSC-CMs. The mRNA-LNP-derived iPSC-CMs presented sarcomeric structures, established an electrically coupled syncytium, and generated field potentials and ventricular-like action potentials similarly to those produced by Sendai virus-differentiated iPSC-CMs (Figure 4). Importantly, both iPSC populations carried over the *KCNH2* variant (Figure 2C,D), and their differentiated iPSC-CMs recapitulated the signature prolonged APD characteristic of the LQTS phenotype (Figure 4D) [29,30,31], indicating that the reprogramming method did not alter the ability of the differentiated iPSC-CMs to manifest the underlying patient-specific electrophysiological phenotype (Figure 4C,D).

As iPSC technology moves toward clinical translation, the methods used to reprogram parental lines are being extensively explored for their suitability in regenerative and precision medicine [46]. In this study, we assessed an mRNA-LNP strategy, a virus-free approach leveraging receptor-mediated endocytosis for cellular uptake [25,47,48]. As a non-viral platform, mRNA-LNP technology mitigates the regulatory, manufacturing, and viral-clearance considerations associated with vector-based systems [49,50]. Unlike Sendai virus-based reprogramming, mRNA-LNP does not introduce residual viral RNA that must be cleared through passaging, preserving those early, high-quality passages for downstream biobanking [46,51], while decreasing workflow timelines. By shortening these timelines, this approach decreased demands for media, consumables, and labor effort, ultimately lowering costs. On a broader scale, such workflow efficiencies could support the scalable iPSC biobanking and biomanufacturing required for future precision-medicine applications and regenerative-medicine pipelines.

Taken together, this study provides a comparative assessment of the mRNA-LNP reprogramming method alongside the established Sendai virus-based method. By utilizing both approaches to reprogram PBMCs from the same donor, we established a genetically identical starting point. This internal control was critical for evaluating the pluripotency of the resulting iPSCs and the functional performance of the derived iPSC-CMs directly against their Sendai virus-derived counterparts. This design ensures that any observed outcomes are attributable to the reprogramming method rather than inter-individual genetic variability. We specifically selected a donor with long QT syndrome because the pronounced and quantifiable electrophysiological signature of this disease, specifically prolonged action potential duration, serves as a clear benchmark to verify that the patient’s phenotype is recapitulated through the mRNA-LNP reprogramming and differentiation process. This choice allowed us to determine whether the distinct pathological signature, as captured by the Sendai virus-derived line, could be equally recovered via the mRNA-LNP approach. Our findings demonstrate that the mRNA-LNP-derived iPSC-CMs successfully recapitulated this signature, mirroring the results of the Sendai virus-derived iPSC-CMs. While this work highlights the technical feasibility and workflow advantages of mRNA-LNP reprogramming, further validation across a broader range of donor backgrounds and genetic pathologies will be essential to fully define the versatility of this platform, as well as to account for natural variation in reprogramming efficiency across individuals, with the present findings derived from the reprogramming of PBMCs from an individual LQTS donor using these two distinct methods. Overall, these findings demonstrate the potential for the mRNA-LNP platform to offer a practical strategy for biorepository biobanking, with the potential to support future applications in regenerative medicine, precision medicine, and disease modeling.

## 4. Materials and Methods

### 4.1. PBMCs Isolation

PBMCs were isolated using SepMate^TM^-50 (IVD) tubes (STEMCELL Technologies, Vancouver, BC, USA) along with Histopaque^TM^ 1077 (Sigma-Aldrich, St. Louis, MO, USA) and maintained in StemPro^TM^-34 SFM medium (Thermo Fisher Scientific, Waltham, MA, USA) containing SCF, FLT3, IL-3, IL-6, GlutaMAX^TM^, and EPO (STEMCELL Technologies) in a 37 °C and 5% CO_2_ incubator for 4 days.

### 4.2. Sendai Virus iPSC Reprogramming

The CytoTune^TM^-iPS 2.0 Sendai Reprogramming Kit (Thermo Fisher Scientific, cat#: A16517) was used for PBMC reprogramming. KOS and c-Myc vectors were used at a 5:5 ratio, and KLF4 was delivered at an MOI of 4.5 to improve reprogramming efficiency. Cells were cultured on Matrigel^®^-coated plates (Corning, Corning, NY, USA) in Essential 8^TM^ Flex medium (Thermo Fisher Scientific). The iPSC colonies were then manually picked and cultured in Essential 8^TM^ Flex medium supplemented with CloneR^TM^2 (STEMCELL Technologies). For the first ten passages, colonies were manually selected, after which cells were passaged as aggregates every 4–5 days at a 1:6 ratio using ReLeSR and CloneR^TM^2.

### 4.3. mRNA-LNP iPSC Reprogramming

iPSCs were generated from PBMCs using the Ubri-iPSC RNA-LNP Reprogramming Kit (Ubrigene, cat#: iPSC-RPM-RNA-LNP-V2, Germantown, MD, USA), utilizing mRNA for five reprogramming factors (Oct4, SOX2, KLF4, c-Myc, and LIN28) encapsulated in lipid nanoparticles (LNPs). Reprogramming was performed according to the manufacturer’s instructions. PBMCs were cultured with Enhancer B and the mRNA-LNP cocktail for two days, then plated onto Laminin 521-coated plates. Three additional mRNA-LNP treatments were given every two days. Cells were then kept in ReproTeSR™ medium (STEMCELL Technologies). Between days 16 and 20, colonies were picked and expanded in Essential 8™ Flex Medium (Thermo Fisher Scientific). iPSCs were passaged using ReLeSR™ (STEMCELL Technologies) and frozen in CryoStor^®^ CS10 (STEMCELL Technologies).

### 4.4. Sanger Sequencing

DNA from PBMCs and iPSCs was extracted using the QIAamp DNA Mini kit (QIAGEN, Hilden, Germany). The *KCNH2* c.2398+5G>T (intronic) (rs1554425149) region was amplified using Platinum^TM^ SuperFi II PCR Master Mix (Invitrogen, Carlsbad, CA, USA). After gel electrophoresis verification, the PCR product was purified with the QIAquick PCR Purification kit (QIAGEN), and Sanger sequenced at Azenta using the forward primer to determine genotype.

### 4.5. Flow Cytometry

Accutase (Thermo Fisher Scientific) was used to dissociate iPSCs, followed by blocking with FcR Blocking Reagent (Miltenyi Biotec, Bergisch Gladbach, Germany). Cells were incubated with surface antibodies (Table S1) for 15–30 min, then fixed and permeabilized using the Inside Stain Kit (Miltenyi Biotec). Cells were incubated with intracellular antibodies for 30–45 min. Analysis was conducted using FACSymphony™ A3 and FlowJo^TM^ software version 10 (BD Biosciences, Franklin Lakes, NJ, USA).

### 4.6. Immunocytochemistry

iPSCs were plated on Matrigel^®^-coated chamber slides, fixed with 4% paraformaldehyde for 20 min, permeabilized using 0.1% Triton X-100 (Sigma-Aldrich) for 10 min, and blocked with donkey serum (Jackson ImmunoResearch, West Grove, PA, USA) for 1 h. Primary antibodies for iPSCs (Table S1) were added and incubated overnight at 4 °C. Secondary antibodies (Table S1) were applied for 1 h at room temperature. EverBrite^TM^ Hardset Mounting Medium with DAPI (Biotium, Fremont, CA, USA) was mounted onto the coverslips and left to cure at room temperature for 1 h. To observe sarcomeres, ventricular cardiomycoytes grown on chamber slides for 2 weeks in maintenance media were incubated with Troponin T antibody overnight, then the anti-rabbit Alexa 568 secondary antibody was added. Images were captured using a ZEISS LSM 900 confocal microscope (Carl Zeiss AG, Oberkochen, Germany).

### 4.7. iPSC Trilineage Differentiation

Matrigel^®^-coated plates were used to plate iPSCs and incubated at 37 °C and 5% CO_2_ for 5–7 days with daily medium replacement using the STEMdiff^TM^ Trilineage Differentiation Kit (STEMCELL Technologies). Flow cytometry was used to assess differentiation into endoderm (CXCR4/SOX17), mesoderm (Brachyury/NCAM), and ectoderm (Nestin/PAX6) as shown in Table S1.

### 4.8. Mycoplasma Testing

The MycoStrip^®^ Mycoplasma Detection Kit (InvivoGen, San Diego, CA, USA) was used to verify that the iPSCs were mycoplasma-free.

### 4.9. Cytogenetic Analysis (Karyotyping)

20 G-banded metaphase iPSCs underwent karyotype analysis with a resolution range of 375–475 bands (WiCell, Madison, WI, USA).

### 4.10. Short Tandem Repeat (STR Analysis)

PBMCs and iPSCs underwent STR analysis at WiCell using the PowerPlex^®^ 16 HS System (Promega Corporation, Madison, WI, USA).

### 4.11. iPSC-CM Differentiation and Maintenance

iPSCs were differentiated into ventricular-like iPSC cardiomyocytes using the STEMdiff™ Ventricular Cardiomyocyte Differentiation Kit (STEMCELL Technologies) according to the manufacturer’s instructions. iPSC colonies were dissociated into a single-cell suspension and plated onto Matrigel-coated plates. Media was changed every two days, and spontaneous contractions were seen by day 8. Cells were then kept in STEMdiff™ Cardiomyocyte Maintenance Medium (STEMCELL Technologies) until day 15, when they were dissociated for immunocytochemistry (ICC) and multielectrode array plating.

### 4.12. Microelectrode Array Plating and Recording

iPSC-CMs were dissociated using STEMdiff™ Cardiomyocyte Dissociation Media (STEMCELL Technologies) and plated onto Axion BioSystems MEA plates directly over each well’s recording electrode array. Following plating, cells were incubated at 37 °C in 5% CO_2_. For the initial 24 h, cells were suspended in STEMdiff™ Cardiomyocyte Support Medium (STEMCELL Technologies) containing STEMdiff™ Cardiomyocyte Plating Supplement (STEMCELL Technologies), in accordance with the manufacturer’s instructions. Thereafter, the media was replaced with STEMdiff™ Cardiomyocyte Maintenance Medium (STEMCELL Technologies) every 48 h. MEA recordings were performed 14 days post-plating using the Maestro Edge™ (Axion BioSystems, Atlanta, GA, USA) under standard cardiac settings (37 °C, 5% CO_2_). Field potentials, signal-propagation maps, and action potentials, recorded via the Local Extracellular Action Potential assay, were recorded using Axion Maestro AxIS Navigator software version 3.10.1. Data analysis and representative tracings were generated using AxIS Navigator, the Axion Data Export Tool, and a custom-written MATLAB (version 24.2) program.

## Figures and Tables

**Table 1:** Comparison of Sendai virus and mRNA-LNP reprogramming and characterization workflow milestones.

Procedure	Reprogramming Using Sendai Virus	Reprogramming Using mRNA-LNP
Passage by colony picking	Up to P10	Up to P2
Morphology and Pluripotency Characterization	As early as P11	As early as P3
Genomic Characterization	As early as P11	As early as P4
Expansion	P10	P2

## Data Availability

The data supporting the conclusions of this article will be made available on request from the corresponding author.

## Supplementary Materials

The following supporting information can be downloaded at: https://www.mdpi.com/article/10.3390/ijms27083588/s1.

## Abbreviations

The following abbreviations are used in this manuscript:

iPSC	Induced pluripotent stem cell
iPSC-CM	iPSC-derived cardiomyocytes
mRNA-LNP	mRNA-lipid nanoparticles
PBMC	Peripheral blood mononuclear cell
LQTS	Long QT syndrome
LQT2	Long QT syndrome type 2
APD	Action potential duration
MEA	Multielectrode array
LEAP	Local Extracellular Action Potential
APD90	APD at 90% repolarization
cTnT	Cardiac Troponin T
LNP	Lipid nanoparticle
ICC	Immunocytochemistry
