# Supplementary material for: mRNA-Lipid Nanoparticle-Mediated Reprogramming and Standard Sendai Virus Reprogramming: Generation of iPSCs and iPSC-Derived Cardiomyocytes
Source: Int J Mol Sci. 2026 Apr 17;27(8):3588. doi: 10.3390/ijms27083588 (PMC13115868; doi:10.3390/ijms27083588)
Supplement: Supplementary file 1 [file ijms-27-03588-s001.zip › Table S1. Reagent specifications.pdf]

Table S1. Reagent specifications

Application	Markers	Dilution	Supplier	Catalog Number	RRID
**Immunocytochemistry – pluripotency markers**	Oct4	1:200	Cell Signaling Technology	2750S	AB_823583
	SSEA4	1:250	Cell Signaling Technology	4755S	AB_1264259
	TRA-1-81	1:250	Millipore	MAB4381	AB_177638
	SOX2	1:250	R and D Systems	AF2018	AB_355110
**Flow cytometry – pluripotency markers**	TRA-1-60-PE	1:50	Miltenyi Biotec	130-122-965	AB_2801990
	SSEA4-APC	1:50	Miltenyi Biotec	130-126-000	AB_2876944
	OCT4-APC	1:50	Miltenyi Biotec	130-117-821	AB_2784445
	SOX2-FITC	1:50	Miltenyi Biotec	130-120-790	AB_2784459
**Flow cytometry – trilineage differentiation markers**	CXCR4-PE	1:20	STEMCELL Technologies	60089	AB_2936358
	SOX17-APC	1:10	R and D Systems	IC1924A	AB_1964715
	NCAM-FITC	1:20	STEMCELL Technologies	60021	AB_2891082
	Brachyury-APC	1:10	R and D Systems	IC2085A	AB_2891298
	Nestin-FITC	1:30	Thermo Fisher Scientific	MA523671	AB_2608687
	PAX6-PE	1:50	Miltenyi Biotec	130-123-311	AB_2819471

**Primary antibodies**	Anti-Cardiac Troponin T antibody	1:200	Abcam	ab209813	AB_2938619
	EverBrite™ Mounting Medium with DAPI	N/A	Biotium	23002	N/A
**Secondary antibodies**	Donkey anti-Mouse IgG (H+L) Alexa Fluor 488	1:250	Invitrogen	A21202	AB_141607
	Donkey anti-Rabbit IgG (H+L) Alexa Fluor 568	1:250	Invitrogen	A10042	AB_2534017
	Goat anti-Rabbit IgG (H+L) Alexa Fluor 568	1:250	Invitrogen	A11011	AB_143157
	Cy™3 AffiniPure® Donkey anti-Goat IgG (H+L)	1:400	Jackson ImmunoResearch Labs	705-165-147	AB_2307351
